# Accurate Channel Estimation and Adaptive Underwater Acoustic Communications Based on Gaussian Likelihood and Constellation Aggregation

**DOI:** 10.3390/s22062142

**Published:** 2022-03-10

**Authors:** Liang Wang, Peiyue Qiao, Junyan Liang, Tong Chen, Xinjie Wang, Guang Yang

**Affiliations:** 1 College of Marine Technology, Ocean University of China, Qingdao 266100, China; wanglianger@ouc.edu.cn; 2 School of Information and Control Engineering, Qingdao University of Technology, Qingdao 266525, China; ljy15290436608@163.com (J.L.); wangxinjie@qut.edu.cn (X.W.); edit231@163.com (G.Y.); 3 School of Marine Science and Technology, Northwestern Polytechnical University, Xi’an 710129, China; ct8690@163.com; 4 School of Electrical and Electronics Engineering, Nanyang Technological University, Singapore 639798, Singapore

**Keywords:** time-varying underwater acoustic channels, superimposed training, Gaussian likelihood, constellation aggregation, turbo equalization

## Abstract

Achieving accurate channel estimation and adaptive communications with moving transceivers is challenging due to rapid changes in the underwater acoustic channels. We achieve an accurate channel estimation of fast time-varying underwater acoustic channels by using the superimposed training scheme with a powerful channel estimation algorithm and turbo equalization, where the training sequence and the symbol sequence are linearly superimposed. To realize this, we develop a ‘global’ channel estimation algorithm based on Gaussian likelihood, where the channel correlation between (among) the segments is fully exploited by using the product of the Gaussian probability-density functions of the segments, thereby realizing an ideal channel estimation of each segment. Moreover, the Gaussian-likelihood-based channel estimation is embedded in turbo equalization, where the information exchange between the equalizer and the decoder is carried out in an iterative manner to achieve an accurate channel estimation of each segment. In addition, an adaptive communication algorithm based on constellation aggregation is proposed to resist the severe fast time-varying multipath interference and environmental noise, where the encoding rate is automatically determined for reliable underwater acoustic communications according to the constellation aggregation degree of equalization results. Field experiments with moving transceivers (the communication distance was approximately 5.5 km) were carried out in the Yellow Sea in 2021, and the experimental results verify the effectiveness of the two proposed algorithms.

## 1. Introduction

Underwater acoustic communication technology can be widely applied in many fields, such as marine pollution monitoring, underwater rescue, underwater autonomous underwater vehicle (AUV) positioning and navigation. However, underwater acoustic channels are characterized by a time-varying multipath. In particular, when there is relative motion between the transceivers, the channel will change rapidly, resulting in fast time-varying multi-path interference, which distorts the received signal waveform and leads to a reduction in or even failure of the decoding performance of the underwater acoustic communication system [1,2,3].

**Figure 1:**
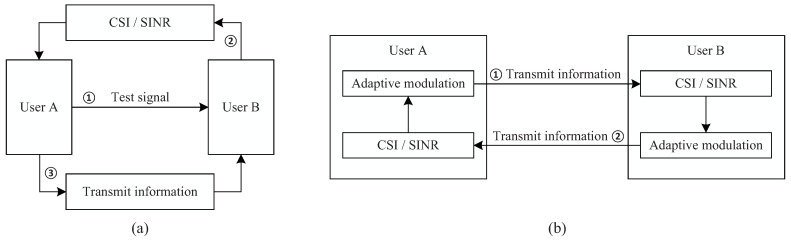
(**a**) Feedback adaptive communications; (**b**) direct adaptive communications.

To solve the issues of time-varying underwater acoustic channels and environmental noise, an adaptive communication scheme was proposed, where the transmitter automatically selected an appropriate modulation according to the instantaneous channel state information (CSI) and signal noise ratio (SNR). The adaptive communication scheme can be mainly divided into two categories, including feedback adaptive communications and direct adaptive communications, as shown in Figure 1a,b, respectively. For feedback adaptive communications, as shown in Figure 1a, User A sends a test signal to User B. User B estimates CSI and SNR based on the test signal, and then feeds them back to User A. User A selects a modulation according to the feedback CSI and SNR, and then transmits the data information to User B by using the selected modulation [4,5]. For direct adaptive communications in Figure 1b, User A initially selects a modulation, such as the direct sequence spread spectrum (DSSS), then transmits the data information to User B by using DSSS. User B identifies the modulation (i.e., identifies DSSS), demodulates and decodes, and estimates CSI and SNR, such as a simple channel and SNR = 20 dB. According to the estimated CSI and SNR, User B selects a new modulation, such as orthogonal frequency division multiplexing (OFDM), and then feeds data information back to User A by using OFDM. Similarly, User A identifies, demodulates and decodes, and estimates CSI and SNR. Then, according to the estimated CSI and SNR, User A selects an original or new modulation, and then transmits the data information to User B by using the selected modulation [6,7]. The biggest difference in the two adaptive communications is that there is no need to send a test signal for the second scheme. Therefore, for a specified amount of data information, the second scheme saves communication time, thereby reducing or even avoiding time-variation of the channel during communications. Therefore, the second scheme is more suitable for fast time-varying channels incurred by underwater acoustic communications with moving transceivers than the first scheme.

For adaptive communications, there are mainly four selected modulations and demodulations: multiple frequency shift keying (MFSK), spread spectrum, orthogonal frequency division multiplexing (OFDM) and single carrier. The transmission rate of MFSK is low; spread spectrum technology always uses high-order spread spectrum code, which has a low communication efficiency [8]; OFDM has a poor anti-frequency-offset performance [9,10,11,12,13,14,15,16]. Therefore, with a high transmission rate and good anti-frequency-offset characteristics [17,18,19], the single carrier technology is adopted in this paper. It can be used with a variety of encoding rates to realize adaptive underwater acoustic communications with moving transceivers.

Channel estimation is one of the key factors to realize reliable adaptive communications. At present, there are mainly three kinds of underwater acoustic channel estimation algorithms, such as channel estimation algorithms based on reference signal, blind estimation algorithms and semi-blind estimation algorithms [20,21,22,23,24,25,26,27,28]. Among the three kinds of algorithms, the channel estimation capability and channel tracking capability based on the reference signal are the strongest. Much research on them has been conducted by some teams, such as the team of the University of Connecticut, the team of the Massachusetts Institute of Technology, the team of Institute of Acoustics, Chinese Academy of Sciences, and the team of Harbin Engineering University. So far, all of the above channel estimation algorithms based on reference signals have adopted the traditional time-multiplexing training sequence scheme. In order to further improve the tracking capability of time-varying channels, the joint team of Qingdao University of Technology, the University of Wollongong and the University of Western Australia [29] proposed a superimposed training scheme for underwater acoustic communications, where the training sequence and the symbol sequence are linearly superimposed in order to make the channel information of the training sequence and the symbol sequence completely consistent.

Same as literatures [29,30], the superimposed training (ST) scheme and the segment strategy are used in this paper to enhance the estimation and tracking capability of fast time-varying channels. To realize the full potential of the ST scheme and the segment strategy, a channel estimation algorithm based on Gaussian likelihood (GL) is proposed. The product of the Gaussian probability-density functions of the segments is still the Gaussian probability-density function, which can be parameterized by the mean and the variance, where the mean is the channel estimate and the variance is the deviation of the channel estimate. The variance of the Gaussian probability-density function after the product is less than the variance of the Gaussian probability-density function of each segment, which means that the estimated channel for the segment after the product is more accurate than the estimated channel for the segment itself. This is equivalent to estimating the channel information of the segment by using the ‘whole’ data block [29,30], thereby leading to the ideal channel estimation of the segment.

It is important to note that the proposed GL algorithm can achieve the same channel estimation and tracking performance as literatures [29,30] in a ‘novel’ Gaussian product way, because it can be seen as a message-passing method in the Gaussian scenario [29,30]. The message-passing thought was first proposed in literature [30] to improve the channel estimation capability; then, it was applied in underwater acoustic communications with a communication distance of approximately 1 km [29]. Different from literatures [29,30], in this paper, the same thought as message passing [29,30] is realized in a ‘novel’ Gaussian product way; in particular, the proposed algorithm is applied in actual underwater acoustic communication machines, and the effective communication distance is extended from 1 km to 5.5 km.

In addition, an adaptive communication algorithm based on constellation aggregation (CA) is proposed. The encoding rate (such as rate-1/2, rate-1/4, rate-1/8, or rate-1/16) is automatically selected based on the aggregation degree of the constellation points after linear minimum mean square error (LMMSE) equalization. The working principles of the proposed direct adaptive communications and the traditional direct adaptive communications are different. The traditional direct adaptive communications select the modulation based on CSI and SNR. However, the proposed direct adaptive communications select the encoding rate based on the constellation aggregation. The proposed algorithm based on the constellation aggregation is more accurate in making a selection than the traditional algorithm based on CSI and SNR. In order to fully realize the potential of the GL algorithm and the CA algorithm, the single-carrier communication system and turbo equalization are adopted. The channel estimator (GL), constellation aggregation decision maker (CA), equalizer and decoder are combined together, and they are performed jointly in an iterative manner (turbo equalization) to realize an accurate estimation of fast time-varying channels and reliable communications by using the information exchange between the equalizer and the decoder (turbo equalization). Field experiments with moving transceivers (the communication distance was approximately 5.5 km) were carried out in the Yellow Sea in 2021 to verify the effectiveness of the proposed algorithms. The major contributions of this paper are summarized as follows:

(1) A channel estimation algorithm, named GL, is proposed, realizing the same performance as the bidirectional channel estimation algorithm [29,30] in a novel product way of probability-density functions;
(2) An adaptive communication algorithm based on constellation aggregation is proposed to improve the applicability of the system for different environments;
(3) GL-based channel estimation, LMMSE equalization and decoding are iteratively performed (turbo equalization), leading to a significant performance improvement of the whole system;
(4) The proposed algorithms are applied in actual underwater acoustic communication machines to verify their effectiveness.

The remainder of the paper is organized as follows. The system structure is provided in Section 2. Then, a channel estimation algorithm based on Gaussian likelihood and an adaptive communication algorithm based on constellation aggregation are shown in Section 3. Simulations, experiments and the conclusion are presented in Section 4, Section 5 and Section 6, respectively. Throughout the paper, superscripts ${\left[\cdot \right]}^{T_{r}}$ and ${\left[\cdot \right]}^{H}$ represent transpose and conjugate transpose, respectively.

## 2. System Structure

**Figure 2:**
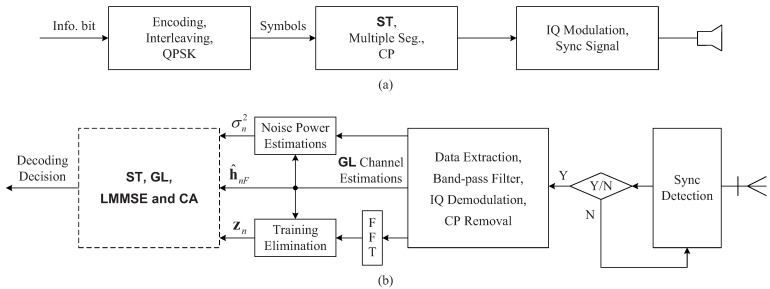
System structure. (**a**) Transmitter; (**b**) receiver.

The system structure of underwater acoustic communications is shown in Figure 2. At the transmitter, the information bit sequence is encoded, interleaved and mapped by using quadrature phase shift keying (QPSK) to symbols. The training sequence and the symbol sequence are linearly superimposed, the resultant sequence is partitioned into multiple segments and then each segment is appended a cyclic prefix (CP) to avoid inter-segment interference and to facilitate low-complexity equalization. The in-phase quadrature (IQ) modulation is used for each CP plus segment. Hyperbolic frequency modulation (HFM) signals with negative and positive modulation rates are used as the head and the tail of the signal frame, respectively. Then, the resultant signals are transmitted by a transducer.

**Figure 3:**
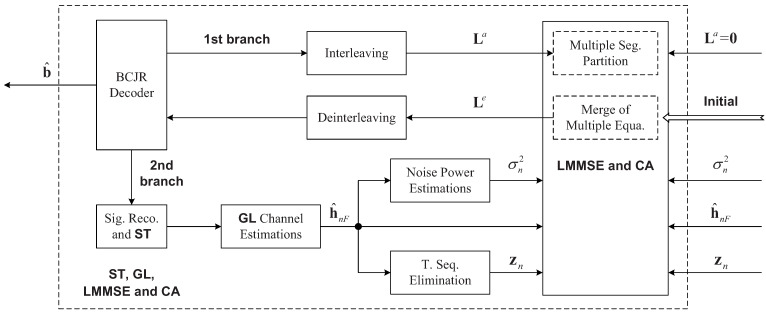
Turbo equalization.

The HFM signals are used to estimate and eliminate the average frequency offset and to synchronize the received signals [31]. Then, the transmitted signals are extracted, band-pass filtering and IQ demodulation are carried out and CPs are removed. With the resultant signals, we estimate initial channels ${\hat{h}}_{nF}$ of all segments based on the GL algorithm and noise powers ${\hat{p}}_{n}$, and obtain a ‘clean’ signal $z_{n}$ after training elimination for data equalization. Then, LMMSE equalization, CA decision and decoding are carried out based on ${\hat{h}}_{nF}$, ${\hat{p}}_{n}$ and $z_{n}$, as shown in Figure 3. They, on both sides of equalization, represent the same things. Please note that, on the right side, they represent the initial values, and, on the left side, they represent the iterative values. The iterative process proceeds until a pre-set number of iterations is reached, and difficult decisions are made on each information bit in the last iteration.

**Figure 4:**
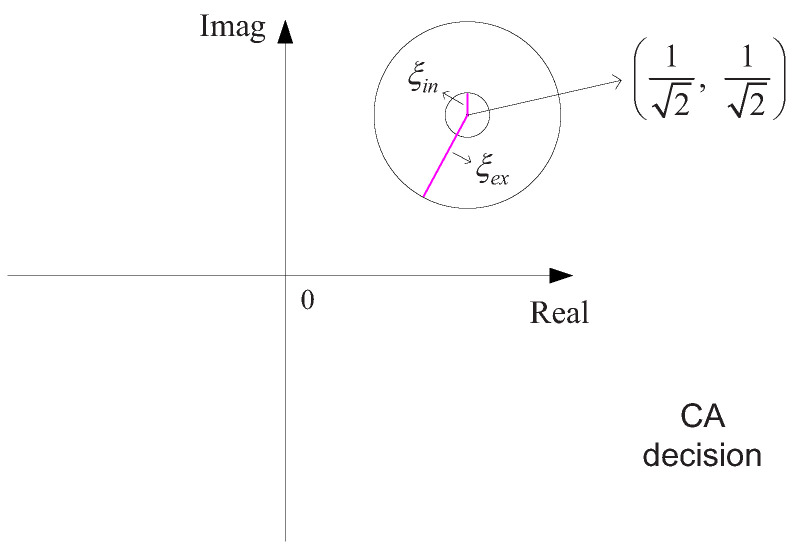
Constellation aggregation decision.

An illustration of the CA decision is shown in Figure 4. We can ensure the return encoding rate by comparing the pre-set values $\xi_{in}$ and $\xi_{ex}$. When the constellation aggregation degree $\xi_{m}<\xi_{in}$, the return encoding rate is increased. When the constellation aggregation degree $\xi_{m}>\xi_{ex}$, the return encoding rate is reduced. When the constellation aggregation degree $\xi_{in}\leq \xi_{m}\leq \xi_{ex}$, the return encoding rate is kept the same.

The turbo equalization is shown in Figure 3. Based on ${\hat{h}}_{nF}$, ${\hat{p}}_{n}$ and $z_{n}$, the LMMSE equalization and decoding for each segment are carried out, where the LMMSE equalization can be efficiently implemented with fast Fourier transform (FFT), where the initial a priori logarithm likelihood ratios (LLRs) of the interleaved encoded bits are set to zeros, i.e., $L^{a}\;=\;0$. The soft detection outputs for multiple segments are collected to make up extrinsic LLRs $L^{e}$, and then deinterleaving and decoding are carried out. The output of the decoder is used by the equalizer and the channel estimator, so there are two branches from the decoder. Both branches use the latest decoding results, i.e., the LLRs of encoded bits of the decoder, and they are updated in each iteration. In the first branch, the LLRs of encoded bits are interleaved and input to the equalizer. In the second branch, difficult decisions on the encoded bits are executed, followed by interleaving and QPSK mapping to obtain the (estimated) symbol sequence. They, together with the training sequence, are used for accurate channel (re)estimation. After that, based on $L^{a}$ from the first branch and ${\hat{h}}_{nF}$, ${\hat{p}}_{n}$ and $z_{n}$ from the second branch, LMMSE equalization is performed to obtain $L^{e}$, which is input into the decoder for the next round of iteration (turbo equalization).

## 3. Accurate Channel Estimation and Adaptive Communications

A block of an information bit sequence denoted by $b\;=\;{\left[b_{1},\cdots ,b_{L_{b}}\right]}^{T_{r}}$ is encoded and interleaved, yielding an interleaved coded bit sequence denoted by $c\;=\;{\left[c_{1},\cdots ,c_{L_{i}}\right]}^{T_{r}}$, where $c_{i}\;=\;{\left[c_{i}^{1},c_{i}^{2}\right]}^{T_{r}}$. Then, c is mapped into a symbol sequence denoted by $f_{L_{f}}\;=\;{\left[f_{1},\cdots ,f_{L_{f}}\right]}^{T_{r}}$, where each $f_{i}$ corresponds to a $c_{i}$. Denote the periodic training sequence as $t_{L_{f}}\;=\;{\left[t_{1},\cdots ,t_{L_{f}}\right]}^{T_{r}}$ with a period of *T*, where $t_{k}\;=\;e^{j\frac{\pi }{T}{\left(k-1\right)}^{2}},\;k\;=\;1,\cdots ,T$ [32]. The training sequence and the symbol sequence are linearly superimposed with a power ratio *r*, yielding the transmitted signal s with length $L_{f}$, where $L_{f}$ is an integer multiple of *T*.

Divide s into $N_{y}$ segments, i.e., $s\;=\;{\left[s_{1},\cdots ,s_{N_{y}}\right]}^{T_{r}}$, and the length of each segment is $L_{s}$, where $L_{f}\;=\;N_{y}\times L_{s}$. Taking $s_{n}$ as an example, the corresponding symbol sequence is $f_{n}$, and the corresponding training sequence is $t_{L_{s}}$. CP is added to each segment, yielding the channel circulant matrix denoted by $H_{n}$. Denote the white Gaussian noise as w.

Denote a segment of the received signal after CP removal as $y_{n}$, and its length is an integer multiple of *T*, i.e., $L_{s}\;=\;pT$. Then, we can represent $y_{n}$ as $y_{n}\;=\;{\left[y_{1T},\cdots ,y_{pT}\right]}^{T_{r}}$. The received signal $y_{n}$ can be written as

(1) $$\begin{array}{cc} y_{n} & =H_{n}s_{n}+w\;=\;H_{n}\left(rt_{L_{s}}+f_{n}\right)+w\\ \; & =H_{n}f_{n}+rH_{n}t_{L_{s}}+w \end{array}.$$

Define $L_{c}$ as the channel order, where $T\geq L_{c}$; then, the Toeplitz matrix formed by the training sequence can be represented as

(2) $$A\;=\;{\left[\begin{array}{cccc} t_{1} & t_{T} & \cdots & t_{T-L_{c}+2}\\ t{}_{2} & t_{1} & \cdots & t_{T-L_{c}+3}\\ \vdots & \vdots & \ddots & \vdots \\ t_{T} & t_{T-1} & \cdots & t_{T-L_{c}+1} \end{array}\right]}_{T\times L_{c}}.$$

From Appendix A, based on the least squares (LS) algorithm, the channel estimate of a segment can be computed as

(3) $${\hat{h}}_{n}\;=\;{\left[{\left(A^{H}A\right)}^{-1}A^{H}\left(\frac{1}{p}\sum_{i\;=\;1}^{p}y_{iT}\right)\right]}_{L_{c}\times 1}.$$

### 3.1. Accurate Channel Estimation Based on Gaussian Likelihood

Channel estimates of two consecutive segments can be expressed as two independent and identically distributed probability-density functions in the Gaussian scenario. Denote two independent and identically distributed probability-density functions as $p_{n-1}\left(x\right)$ and $p_{n}\left(x\right)$. Denote $\mu_{{\hat{h}}_{n-1}}$ and $\sigma_{{\hat{h}}_{n-1}}^{2}$ as the mean value and variance of the channel estimate ${\hat{h}}_{n-1}$ of the (*n*-1)-th segment, respectively, and denote $\mu_{{\hat{h}}_{n}}$ and $\sigma_{{\hat{h}}_{n}}^{2}$ as the mean value and variance of the channel estimate ${\hat{h}}_{n}$ of the *n*-th segment, respectively. Then, we can obtain

(4) $$\left\{\begin{array}{c} p_{n-1}\left(x\right)\;=\;\frac{1}{\sqrt{2\pi }\sigma_{{\hat{h}}_{n-1}}^{}}e^{-\frac{{\left(x-\mu_{{\hat{h}}_{n-1}}\right)}^{2}}{2\sigma_{{\hat{h}}_{n-1}}^{2}}}\\ p_{n}\left(x\right)\;=\;\frac{1}{\sqrt{2\pi }\sigma_{{\hat{h}}_{n}}^{}}e^{-\frac{{\left(x-\mu_{{\hat{h}}_{n}}\right)}^{2}}{2\sigma_{{\hat{h}}_{n}}^{2}}} \end{array}\right..$$

Denote ${\hat{h}}_{nF}$ as the channel estimate after information fusion of the channel estimate ${\hat{h}}_{n-1}$ of the (*n*-1)-th segment and the channel estimate ${\hat{h}}_{n}$ of the *n*-th segment. Denote $\mu_{{\hat{h}}_{nF}}$ and $\sigma_{{\hat{h}}_{nF}}^{2}$ as the mean value and variance of the channel estimate ${\hat{h}}_{nF}$ after information fusion, respectively. Then, the product of the two probability-density functions can be expressed as

(5) $$\begin{array}{cc} p_{n-1}\left(x\right)p_{n}\left(x\right)= & \frac{1}{2\pi \sigma_{{\hat{h}}_{n}}^{}\sigma_{{\hat{h}}_{n-1}}^{}}e^{-\left[\frac{{\left(x-\mu_{{\hat{h}}_{n}}^{}\right)}^{2}}{2\sigma_{{\hat{h}}_{n}}^{2}}+\frac{{\left(x-\mu_{{\hat{h}}_{n-1}}^{}\right)}^{2}}{2\sigma_{{\hat{h}}_{n-1}}^{2}}\right]}\\ = & \left(\frac{1}{\sqrt{2\pi (\sigma_{{\hat{h}}_{n}}^{2}+\sigma_{{\hat{h}}_{n-1}}^{2})}}e^{-\frac{{\left(\mu_{{\hat{h}}_{n}}^{}-\mu_{{\hat{h}}_{n-1}}^{}\right)}^{2}}{2\left(\sigma_{{\hat{h}}_{n}}^{2}+\sigma_{{\hat{h}}_{n-1}}^{2}\right)}}\right)\frac{1}{\sqrt{2\pi \sigma_{{\hat{h}}_{nF}}^{2}}}e^{-\frac{{\left(x-\mu_{{\hat{h}}_{nF}}\right)}^{2}}{2\sigma_{{\hat{h}}_{nF}}^{2}}}\\ = & C_{A}\frac{1}{\sqrt{2\pi \sigma_{{\hat{h}}_{nF}}^{2}}}e^{-\frac{{\left(x-\mu_{{\hat{h}}_{nF}}\right)}^{2}}{2\sigma_{{\hat{h}}_{nF}}^{2}}}, \end{array}$$

where

(6) $$\begin{array}{c} \mu_{{\hat{h}}_{nF}}\;=\;\frac{\mu_{{\hat{h}}_{n}}\sigma_{{\hat{h}}_{n-1}}^{2}+\mu_{{\hat{h}}_{n-1}}\sigma_{{\hat{h}}_{n}}^{2}}{\sigma_{{\hat{h}}_{n}}^{2}+\sigma_{{\hat{h}}_{n-1}}^{2}}, \end{array}$$

and

(7) $$\begin{array}{c} \sigma_{{\hat{h}}_{nF}}^{2}\;=\;\frac{\sigma_{{\hat{h}}_{n}}^{2}\sigma_{{\hat{h}}_{n-1}}^{2}}{\sigma_{{\hat{h}}_{n}}^{2}+\sigma_{{\hat{h}}_{n-1}}^{2}}. \end{array}$$

It is important to note that

(8) $$\left\{\begin{array}{c} \sigma_{{\hat{h}}_{nF}}^{2}-\sigma_{{\hat{h}}_{n-1}}^{2}\;=\;\frac{\sigma_{{\hat{h}}_{n}}^{2}\sigma_{{\hat{h}}_{n-1}}^{2}}{\sigma_{{\hat{h}}_{n}}^{2}+\sigma_{{\hat{h}}_{n-1}}^{2}}-\sigma_{{\hat{h}}_{n-1}}^{2}\;=\;\frac{-\sigma_{{\hat{h}}_{n-1}}^{4}}{\sigma_{{\hat{h}}_{n}}^{2}+\sigma_{{\hat{h}}_{n-1}}^{2}}<0\\ \sigma_{{\hat{h}}_{nF}}^{2}-\sigma_{{\hat{h}}_{n}}^{2}\;=\;\frac{\sigma_{{\hat{h}}_{n}}^{2}\sigma_{{\hat{h}}_{n-1}}^{2}}{\sigma_{{\hat{h}}_{n}}^{2}+\sigma_{{\hat{h}}_{n-1}}^{2}}-\sigma_{{\hat{h}}_{n}}^{2}\;=\;\frac{-\sigma_{{\hat{h}}_{n}}^{4}}{\sigma_{{\hat{h}}_{n}}^{2}+\sigma_{{\hat{h}}_{n-1}}^{2}}<0 \end{array}\right.,$$

which means that the variance $\sigma_{{\hat{h}}_{nF}}^{2}$ after the product becomes smaller than $\sigma_{{\hat{h}}_{n-1}}^{2}$ and $\sigma_{{\hat{h}}_{n}}^{2}$, i.e., the fused channel estimate ${\hat{h}}_{nF}$ becomes more accurate, which is more close to the real channel than ${\hat{h}}_{n-1}$ and ${\hat{h}}_{n}$. $C_{A}$ is the scale factor of the Gaussian distribution, and it is not a variable, which can be normalized. Therefore, we can obtain the Gaussian distribution $p_{nF}\left(x\right)$ after the product, i.e.,

(9) $$p_{n-1}\left(x\right)p_{n}\left(x\right)\;=\;p_{nF}\left(x\right)∼N\left(\mu_{{\hat{h}}_{nF}},\sigma_{{\hat{h}}_{nF}}^{2}\right),$$

where $N\left(\right)$ represents Gaussian distribution. From (7), we can acquire

(10) $$\frac{1}{\sigma_{{\hat{h}}_{nF}}^{2}}\;=\;\frac{\sigma_{{\hat{h}}_{n}}^{2}+\sigma_{{\hat{h}}_{n-1}}^{2}}{\sigma_{{\hat{h}}_{n}}^{2}\sigma_{{\hat{h}}_{n-1}}^{2}}\;=\;\frac{1}{\sigma_{{\hat{h}}_{n-1}}^{2}}+\frac{1}{\sigma_{{\hat{h}}_{n}}^{2}}.$$

From (6) and (7), i.e., $\mu_{{\hat{h}}_{nF}}$ is divided by $\sigma_{{\hat{h}}_{nF}}^{2}$, we can obtain

(11) $$\frac{\mu_{{\hat{h}}_{nF}}}{\sigma_{{\hat{h}}_{nF}}^{2}}\;=\;\frac{\mu_{{\hat{h}}_{n}}\sigma_{{\hat{h}}_{n-1}}^{2}+\mu_{{\hat{h}}_{n-1}}\sigma_{{\hat{h}}_{n}}^{2}}{\sigma_{{\hat{h}}_{n-1}}^{2}\sigma_{{\hat{h}}_{n}}^{2}}\;=\;\frac{\mu_{{\hat{h}}_{n-1}}}{\sigma_{{\hat{h}}_{n-1}}^{2}}+\frac{\mu_{{\hat{h}}_{n}}}{\sigma_{{\hat{h}}_{n}}^{2}},$$

i.e.,

(12) $$\mu_{{\hat{h}}_{nF}}\;=\;\sigma_{{\hat{h}}_{nF}}^{2}\left(\frac{\mu_{{\hat{h}}_{n-1}}}{\sigma_{{\hat{h}}_{n-1}}^{2}}+\frac{\mu_{{\hat{h}}_{n}}}{\sigma_{{\hat{h}}_{n}}^{2}}\right).$$

The message fusion Formulas (10) and (12) are equivalent to the message fusion Formulas (18) and (19) of literature [29], i.e., the proposed GL algorithm using a ‘novel’ Gaussian product can achieve the same performance as the bidirectional channel estimation algorithm in literature [29]. They have the same computational complexity.

The formulas of the forward passing and the backward passing are as follows [33]:

(13) $$\left\{\begin{array}{c} {\hat{h}}_{n}\;=\;\alpha_{p}{\hat{h}}_{n-1}+n_{p}\\ \sigma_{{\hat{h}}_{n}}^{2}\;=\;\alpha_{p}^{2}\sigma_{{\hat{h}}_{n-1}}^{2}+\beta I \end{array}\right.,$$

and

(14) $$\left\{\begin{array}{c} {\hat{h}}_{n-1}\;=\;\alpha_{p}^{-1}({\hat{h}}_{n}+n_{p})\\ \sigma_{{\hat{h}}_{n-1}}^{2}\;=\;\alpha_{p}^{-2}\left(\sigma_{{\hat{h}}_{n}}^{2}+\beta I\right), \end{array}\right.$$

where $\alpha_{p}$ is the channel correlation coefficient of the consecutive segments, $n_{p}$ is Gaussian white noise (the mean is 0) and β is the noise power.

**Figure 5:**
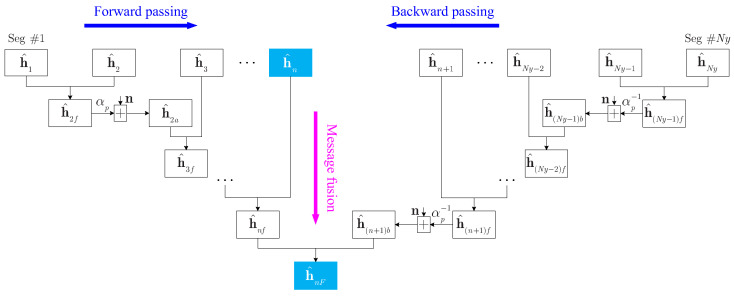
Accurate channel estimation of the *n*-th segment.

Take the *n*-th segment as an example to show the flow of the ‘global’ channel estimation, and the flow diagram is shown in Figure 5. For forward message passing, the local channel estimation ${\hat{h}}_{1}$ of the first segment is fused with the local channel estimation ${\hat{h}}_{2}$ of the second segment to obtain a fused channel estimation ${\hat{h}}_{2f}$ by using (10) and (12). Then, the message update ${\hat{h}}_{2a}$ can be obtained by using (13) until the fused channel estimation ${\hat{h}}_{nf}$ is acquired. For backward message passing, the local channel estimation ${\hat{h}}_{N_{y}}$ of the last segment is fused with the local channel estimation ${\hat{h}}_{N_{y}-1}$ of the ($N_{y}$-1)-th segment to obtain a fused channel estimation ${\hat{h}}_{\left(N_{y}-1\right)f}$ by using (10) and (12). Then, the message update ${\hat{h}}_{\left(N_{y}-1\right)b}$ can be obtained by using (14) until the fused channel estimation ${\hat{h}}_{(n+1)b}$ is acquired. Finally, ${\hat{h}}_{nf}$ and ${\hat{h}}_{(n+1)b}$ can be fused to obtain a ‘global’ channel estimation ${\hat{h}}_{nF}$ of the *n*-th segment. Appending a proper number of zeros to ${\hat{h}}_{nF}$ to form a length-$L_{s}$ vector, i.e., ${\hat{h}}_{nF}\;=\;{\left[{\hat{h}}_{nF},0\right]}_{L_{s}\times 1}$.

### 3.2. Training Interference Elimination, Estimation of Noise Power and Turbo Equalization

We use F to denote a normalized discrete Fourier transform (DFT) matrix, i.e., the (m, n)th element is given as $L_{s}^{-1/2}e^{-j2\pi mn/L_{s}}$ with $j\;=\;\sqrt{-1}$. Take the *n*-th segment as an example. The circulant matrix $H_{n}$ can be diagonalized by a DFT matrix, i.e., $H_{n}\;=\;F^{H}D_{n}F$, where $D_{n}$ is a diagonal matrix. After the training interference elimination, the frequency-domain received signal can be written as

(15) $$\begin{array}{cc} z_{n} & =z_{nf}-F{\hat{h}}_{nF}.*Ft_{L_{s}}\;=\;Fy_{n}-F{\hat{h}}_{nF}.*Ft_{L_{s}}\\ \; & =D_{n}Fs_{n}+\left[D_{n}\left(rFt_{L_{s}}\right)-F{\hat{h}}_{nF}.*Ft_{L_{s}}+Fw_{n}\right]\\ \; & =D_{n}Fs_{n}+{w^{\prime }}_{n} \end{array}.$$

Based on the estimated channel, the diagonal elements of the diagonal matrix ${\hat{D}}_{n}$ can be acquired as follows:

(16) $${\left[{\hat{d}}_{n}^{1},{\hat{d}}_{n}^{2},\cdots ,{\hat{d}}_{n}^{L_{s}}\right]}^{T_{r}}\;=\;\sqrt{L_{s}}F{\hat{h}}_{nF},\;n\;=\;1,\ldots ,N_{y}.$$

As the power of the transmitted symbol sequence is set to 1, the noise power $\sigma_{n}^{2}$ for the *n*-th segment is the difference between the power $Py_{n}$ for the received segment and the corresponding channel energy $E{\hat{h}}_{nF}$, i.e.,

(17) $$\sigma_{n}^{2}\;=\;Py_{n}-E{\hat{h}}_{nF}.$$

Take the *n*-th segment as an example of LMMSE equalization. Following literatures [30,33,34], the a priori mean and variance of the symbol $f_{i}$ (the symbol sequence $f_{n}$) are as follows:

(18) $$\left\{\begin{array}{c} m_{i}^{a}\;=\;\frac{1}{\sqrt{2}}\tanh\left(\frac{L_{n}^{a}\left(c_{i}^{1}\right)}{2}\right)+j\frac{1}{\sqrt{2}}\tanh\left(\frac{L_{n}^{a}\left(c_{i}^{2}\right)}{2}\right)\\ \nu_{i}^{a}\;=\;1-{\left|m_{i}^{a}\right|}^{2} \end{array}\right.,$$

where both the initial values of $L_{n}^{a}\left(c_{i}^{1}\right)$ and $L_{n}^{a}\left(c_{i}^{2}\right)$ (the initial a priori LLRs of the interleaved encoded bits) are set to 0. The estimated interleaved bit sequence is converted to the symbol sequence $m^{a}\;=\;\left[m_{1}^{a},\cdots ,m_{L_{s}}^{a}\right]$ by using (18). The a posteriori mean and variance of the symbol $f_{i}$ (the symbol sequence $f_{n}$) are as follows:

(19) $$\left\{\begin{array}{c} \nu_{1}^{p}\;=\;\nu_{2}^{p}\;=\;\cdots \;=\;\nu_{L_{s}}^{p}\;=\;\frac{1}{L_{s}}\sum_{k\;=\;1}^{L_{s}}{\left(\frac{1}{\bar{v}}+\frac{{\left|{\hat{d}}_{n}^{k}\right|}^{2}}{\sigma_{n}^{2}}\right)}^{-1}\\ m^{p}\;=\;m^{a}+F^{H}{\hat{D}}_{n}^{H}{\left(\frac{\sigma_{n}^{2}}{\bar{v}}I+{\hat{D}}_{n}{\hat{D}}_{n}^{H}\right)}^{-1}\left(z_{n}-{\hat{D}}_{n}Fm^{a}\right) \end{array}\right.,$$

where $\bar{v}\;=\;\frac{1}{L_{s}}\sum_{i\;=\;1}^{L_{s}}v_{i}^{a}$ and $m^{p}\;=\;\left[m_{1}^{p},\cdots ,m_{L_{s}}^{p}\right]$. The a posteriori mean sequence $m^{p}$ is the estimated symbol sequence after LMMSE equalization. It is noted that the computational complexity of the LMMSE equalizer is dominated by (19), and its computational complexity is only in the order of log(Ls) per symbol. In addition, the extrinsic mean and variance of the symbol $f_{i}$ (the symbol sequence $f_{n}$) are as follows:

(20) $$\left\{\begin{array}{c} \nu_{i}^{e}\;=\;{\left(\frac{1}{\nu_{i}^{p}}-\frac{1}{\nu_{i}^{a}}\right)}^{-1}\\ m_{i}^{e}\;=\;\nu_{i}^{e}\left(\frac{m_{i}^{p}}{\nu_{i}^{p}}-\frac{m_{i}^{a}}{\nu_{i}^{a}}\right) \end{array}\right..$$

As QPSK mapping is used, the extrinsic LLRs of the interleaved encoded bits $c_{i}^{1}$ and $c_{i}^{2}$ can be expressed as

(21) Lneci1=22Remie/vieLneci2=22Immie/vie.

The estimated symbol sequence is converted to the extrinsic LLRs (i.e., the estimated interleaved bit sequence $L_{n}^{e}$) by using (19)–(21). The extrinsic LLRs of the segments are denoted collectively as $L^{e}$ and then input into the decoder for the next round of iteration (turbo equalization).

### 3.3. Adaptive Underwater Acoustic Communications Based on Constellation Aggregation

We set a certain iteration number, such as one iteration, to obtain the aggregation degree after LMMSE equalization, i.e., (22), where ${\hat{f}}_{i}$ is the a posteriori mean of a symbol $f_{i}$, and its real part and imaginary part are denoted by f^iRe and f^iIm, respectively.

(22) ξi=f^iRe,f^iIm−1/21,−1,1,1,−1,−1,−1,1=absf^iRe+j×absf^iIm−1/2+j×1/2=absf^iRe−1/22+absf^iIm−1/22.

We compute the mean of all $\xi_{i}$ for a frame of information bits, i.e., $\xi_{m}\;=\;\frac{1}{L_{f}}\sum_{i\;=\;1}^{L_{f}}\xi_{i}$. Denote $\xi_{in}$ and $\xi_{ex}$ as the inner boundary and the outer boundary, respectively. As shown in Figure 4, when $\xi_{m}<\xi_{in}$, the encoding rate will be increased automatically; when $\xi_{m}>\xi_{ex}$, the encoding rate will be reduced automatically; when $\xi_{in}\leq \xi_{m}\leq \xi_{ex}$, the encoding rate will be kept.

## 4. Simulation Results

**Figure 6:**
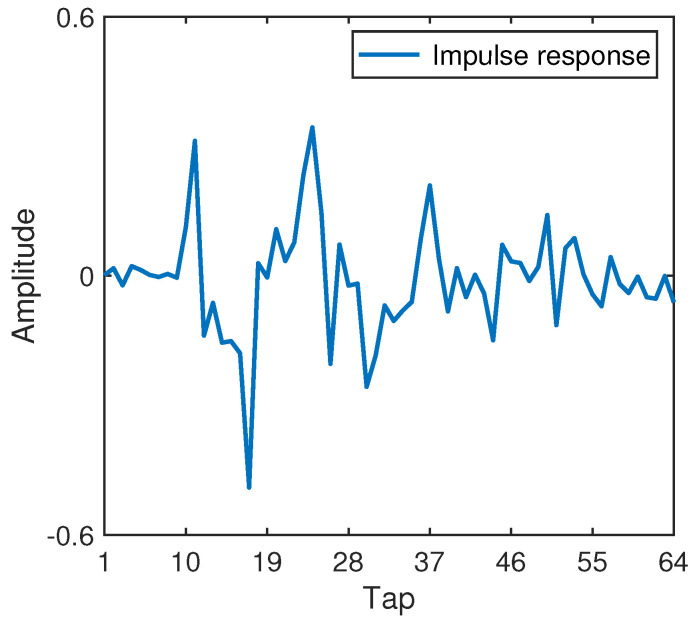
Impulse response of a channel.

The simulation parameters are shown in Table 1. Rate-1/2, rate-1/4, rate-1/8 and rate-1/16 convolutional codes and QPSK mapping are used. A variety of power ratios of the training sequence and the symbol sequence, such as 0.15:1, 0.2:1, 0.25:1 and 0.3:1, are used. The standard block with 1024 symbols is divided into a number of segments with a variety of lengths, including 128 symbols, 256 symbols, 512 symbols and 1024 symbols. The corresponding cases are denoted by S128, S256, S512 and W1024, respectively, where the prefix W means that the standard block is treated as a segment. W1024 is used as the benchmark turbo system. S128, S256 and S512 are used in the proposed GL turbo system. The CP is set to 128 symbols. One frame includes 100 blocks, and one block includes 1024 information bits. Assume that a 4 kHz bandwidth is provided. For S256, with rate-1/2, rate-1/4, rate-1/8 and rate-1/16 convolutional codes, the transmission rates are 2667 bits/s, 1333 bits/s, 667 bits/s and 333 bits/s, respectively, and the corresponding bandwidth efficiencies are 0.67 bps/Hz, 0.33 bps/Hz, 0.17 bps/Hz and 0.08 bps/Hz, respectively. The SNR is from −4 dB to 13 dB. A static channel, as shown in Figure 6, and the white Gaussian noise are used.

**Figure 7:**
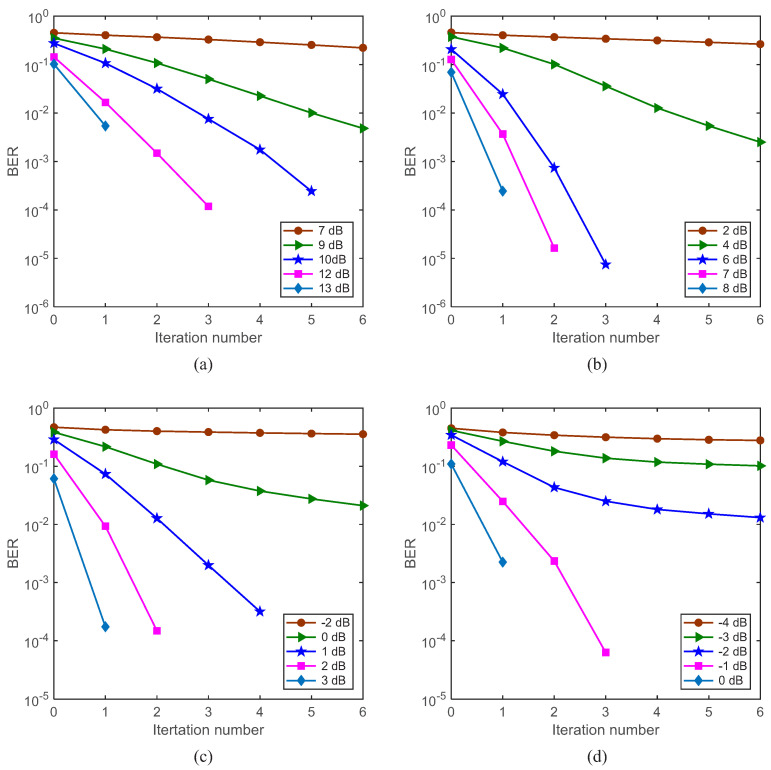
BER performance for S256. (**a**) Rate-1/2 convolutional code; (**b**) rate-1/4 convolutional code; (**c**) rate-1/8 convolutional code; (**d**) rate-1/16 convolutional code.

The BER performance for S256 is shown in Figure 7. It can be seen from the results that both the ST scheme and the channel estimate fusion based on Gaussian likelihood are effective. The lower the encoding rate, the better the BER performance that the system can achieve. Taking Figure 7b, with SNR = 7 dB and rate-1/4 convolutional code, after three iterations, 100 blocks of information bits are correctly decoded. From Figure 7, after two iterations, all information bits with rate-1/2 convolutional code and SNR = 13 dB (Figure 7a), with rate-1/4 convolutional code and SNR = 8 dB (Figure 7b), with rate-1/8 convolutional code and SNR = 3 dB (Figure 7c) and with rate-1/16 convolutional code and SNR = 0 dB (Figure 7d) are correctly decoded.

**Figure 8:**
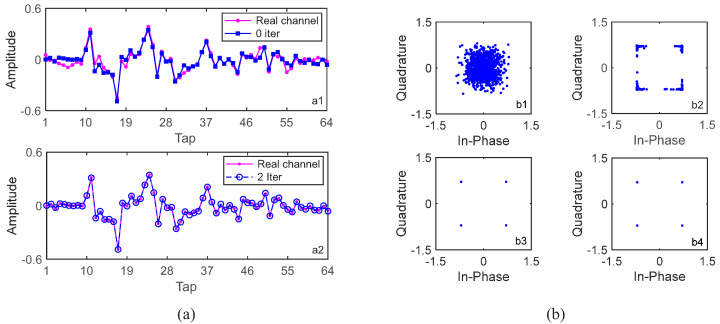
Estimated channels and constellations of a block of 1024 information bits with S256 in simulations at SNR = 13 dB. (**a1**) Channel estimate of one segment without iteration; (**a2**) channel estimate of one segment after 2 iterations; (**b1**) constellations after 0 iteration; (**b2**) constellations after 1 iteration; (**b3**) constellations after 2 iterations; (**b4**) constellations after 3 iterations.

Taking a block of 1024 information bits with rate-1/4 convolutional code as an example, where the noise is not added, the channel in Figure 6 is used, and the channel estimation and equalization results are shown in Figure 8. S256 and a static channel are used; therefore, the channels of the four consecutive segments are the same and their channels are perfectly correlated, i.e., $\alpha_{p}=1$. When turbo equalization is not used, i.e., with 0 iterations, the corresponding channel estimate and equalization results are shown in Figure 8(a1),(b1). The estimated channel in Figure 8(a1) is obviously different from the real channel in Figure 6, and the constellation points after LMMSE equalization in Figure 8(b1) are significantly scattered. When turbo equalization is performed once, i.e., after one iteration, the corresponding equalization results are shown in Figure 8(b2), where it is noted that the estimated channels have been updated before turbo equalization, and the aggregation degree of the constellation points after LMMSE equalization becomes significantly better. When turbo equalization is performed two times, i.e., after two iterations, the corresponding equalization results are shown in Figure 8(b3), where the constellation points after LMMSE equalization are ideally condensed together, and the corresponding estimated channel in Figure 8(a2) is exactly the same as the real channel in Figure 6, demonstrating the effectiveness of the ST scheme used for enhancing the channel estimation and tracking capability and the GL algorithm used for the channel information fusion of the segments.

From Figure 8b, it is clear that we can carry out adaptive communications according to the pre-set constellation aggregation degree threshold. It is important to note that we do not show the adaptive communication performance, as we can see the results clearly from Figure 7.

**Figure 9:**
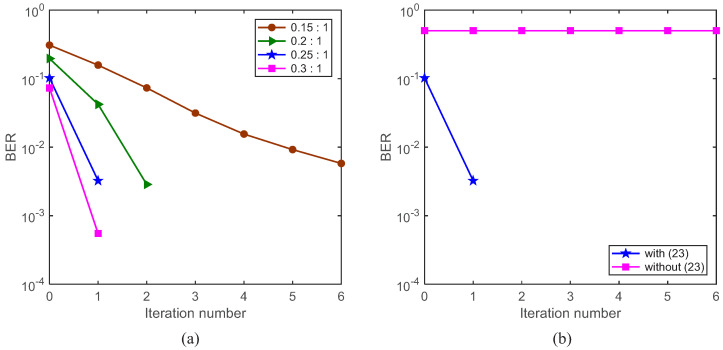
BER performance of the system with S128 and SNR = 13 dB. (**a**) A variety of power ratios of the training sequence and the symbol sequence; (**b**) with or without the training interference elimination. The power ratio is 0.25:1.

Next, we test the BER performance with a variety of power ratios of the training sequence and the symbol sequence. Taking S128 with rate-1/2 convolutional code as an example, the BER performance of the system is shown in Figure 9a. The green triangle line represents the BER performance with a power ratio 0.2:1 and SNR = 13 dB; after three iterations, 100 blocks of information bits are correctly decoded. Considering the complexity and variability of underwater acoustic channels incurred by the moving transceivers, the power ratio 0.25:1 is used in the follow-up simulations and experiments. Assuming that the SNR = 13 dB and the power ratio is 0.25:1, the BER performance of the system is shown in Figure 9b. The blue star line represents the BER performance of the system with the training interference elimination; after two iterations, 100 blocks of information bits are correctly decoded, demonstrating the effectiveness of the training interference elimination. The pink square line represents the BER performance of the system without the training interference elimination, and it can be seen that, if we do not use the training interference elimination, the system simply does not work.

**Figure 10:**
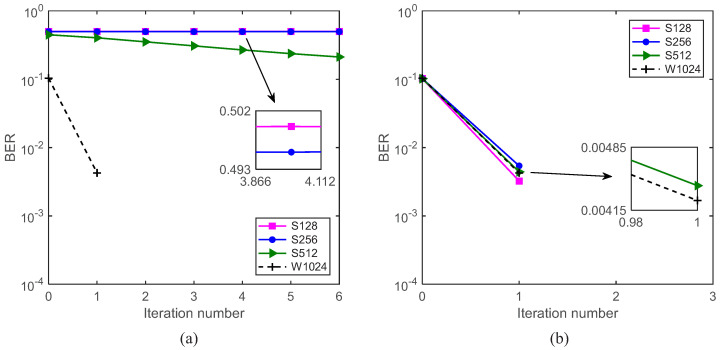
BER performance of the system with SNR = 13 dB, rate-1/2 convolutional code and the power ratio 0.25:1. (**a**) The GL algorithm is not used for channel estimation; (**b**) the GL algorithm is used for channel estimation.

Then, we test the BER performance of the system by using the ST scheme and the GL algorithm, where W1024 is used as the benchmark turbo system. The BER performance comparison is shown in Figure 10. From Figure 10a, if the GL algorithm is not used, the system with a variety of segment lengths does not work. From Figure 10b, if the GL algorithm is used to fuse the local channel estimates to obtain global channel estimates, it can be seen that, no matter how long the segment is, the BER performances for S128, S256, S512 and W1024 are similar. This is because, regardless of the segment length, the ‘whole’ standard symbol block is used to acquire the global channel estimate for each segment. This demonstrates that the proposed GL turbo system (S128, S256 and S512) can achieve a similar performance as the benchmark turbo system (W1024).

## 5. Experimental Results

**Figure 11:**
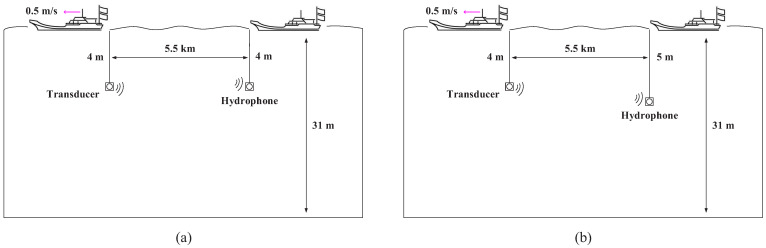
Experimental deployment. (**a**) Yellow Sea 1; (**b**) Yellow Sea 2.

Two separate underwater acoustic communication experiments with moving transceivers were carried out in the Yellow Sea in 2021, named Yellow Sea 1 and Yellow Sea 2, respectively. Their deployments are shown in Figure 11a,b, respectively. We did not use a vertical array in the experiments. The two receiving hydrophones were completely independent, i.e., they had nothing to do with each other. Therefore, multiple receiver channels were not exploited in the system. The height of the sea waves was from 0.5 m to 1 m; the sea temperature was 5.6 °C; the south wind was from level 3 to level 4; the ship with the transducer floated away from the ship with the hydrophone at a speed of approximately 0.5 m/s.

**Figure 12:**
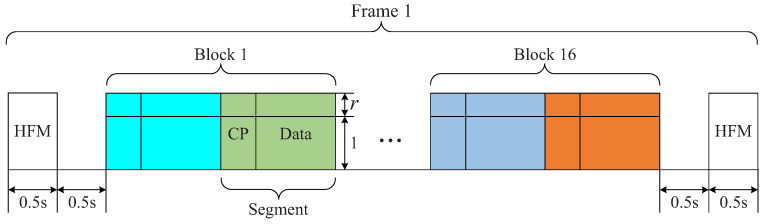
Signal structure for field experiments.

The detailed experimental parameters are shown in Table 1. For the two experiments, QPSK mapping and the power ratio of the training sequence and the symbol sequence 0.25:1 were used; both the communication distances of the transceivers were approximately 5.5 km; one frame included 16 blocks, and one block included 1024 information bits; the single-carrier communication system was used; the center frequency was 12 kHz with a bandwidth of 4 kHz; and the sampling frequency was 96 kHz. The signal structure for field experiments is shown in Figure 12.

### 5.1. Adaptive Underwater Acoustic Communications with SNR = 9 dB

**Figure 13:**
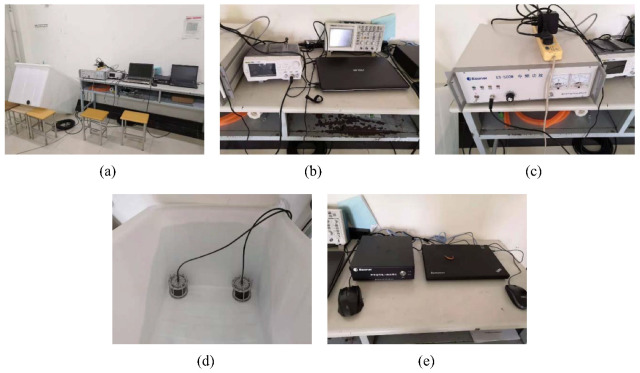
Experimental instruments in the Yellow Sea. (**a**) The whole system; (**b**) transmitter; (**c**) power amplifier; (**d**) the transducer and the hydrophone; (**e**) receiver.

The experimental deployment and instruments for Yellow Sea 1 are shown in Figure 11a and Figure 13, respectively. Rate-1/4, rate-1/8 and rate-1/16 convolutional codes were adopted. S256, S512 and W1024 were used, and the CP was set to 128 symbols. Taking S256, for rate-1/4, rate-1/8 and rate-1/16 convolutional codes, the transmission rate were 1333 bits/s, 667 bits/s and 333 bits/s, respectively, and the corresponding bandwidth efficiencies were 0.33 bps/Hz, 0.17 bps/Hz and 0.08 bps/Hz, respectively. Both transceivers were deployed at a depth of 4 m.

**Figure 14:**
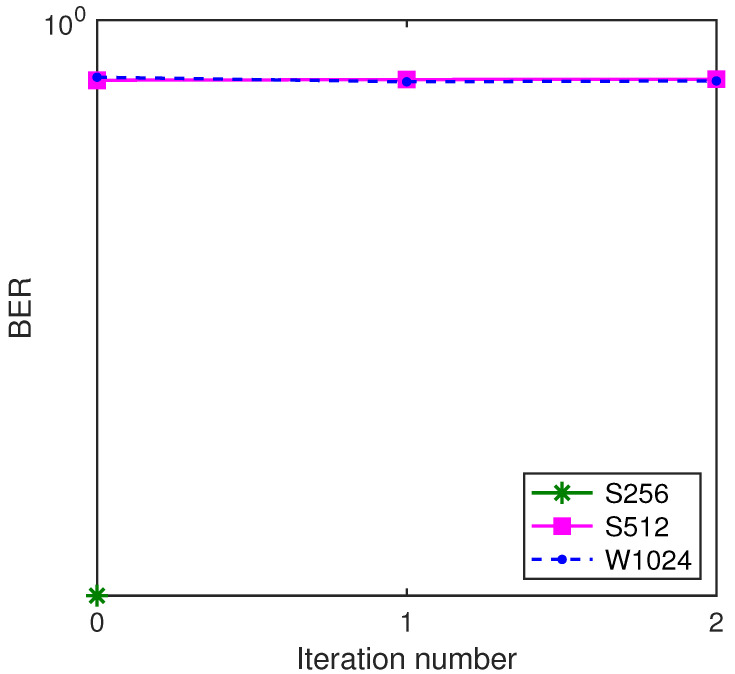
BER performance of the GL turbo system with rate-1/16 convolutional code for Yellow Sea 1.

**Figure 15:**
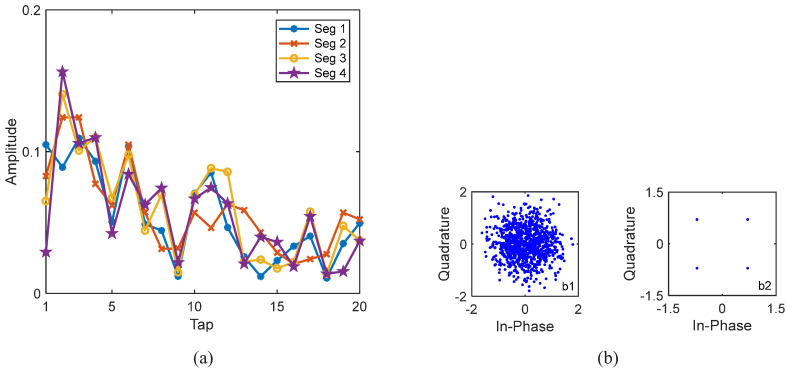
Estimated channels and equalization results of the first block with S256 and 1/16 code rate for Yellow Sea 1 in Figure 14. (**a**) The estimated channels of the consecutive four segments after 1 iteration; (**b1**) constellations after 0 iteration; (**b2**) constellations after 1 iteration.

We firstly used rate-1/16 convolutional code, and the BER performance of 16 data blocks based on the GL algorithm is shown in Figure 14. By comparing the results of S256, S512 and the benchmark turbo system (W1024), it can be seen that S256 was much more effective than S512 and the benchmark turbo system for underwater acoustic communications with moving transceivers. After only one iteration, all information bits with S256 were correctly decoded. However, both S512 (pink square curve) and the benchmark turbo system (blue dotted curve) are completely invalid. This is because moving communications incur time-varying channels. The average channel estimate does not effectively represent the channel information of the 512 symbol block and 1024 symbol block. Taking the first block for S256 in Figure 14 as an example, as S256 was used, there were four consecutive segments for the first data block, and their channels were different due to floating transceivers, i.e., $\alpha_{p}\neq 1$. It is important to note that $\alpha_{p}$ can be obtained automatically, and can be calculated by using the estimated channels of the four segments, as $\alpha_{p}$ was equal to the correlated coefficient of the estimated channels of the four segments, which was also used in the initial channel estimation. When turbo equalization was not used, i.e., with 0 iterations, the corresponding channel equalization results are shown in Figure 15(b1), and the constellation points after LMMSE equalization were very scattered. Then, the automatically determined $\alpha_{p}$ was recalculated by using the updated channel estimates of the four segments. When turbo equalization was performed once, i.e., after one iteration, the corresponding equalization results are shown in Figure 15(b2), where the constellation points after LMMSE equalization were ideally condensed together. The corresponding estimated channels of the four segments in Figure 15a were significantly different, where $\alpha_{p}$ = 0.07 after one iteration, demonstrating the time-variation of the channel and the effectiveness of the ST scheme and the GL algorithm.

Then, we carried out field experiments with a variety of convolutional codes to test the effectiveness of direct adaptive communications. The adaptive threshold setting is shown in Table 2. We used the mean aggregation degree after one iteration to compare the threshold, where the inner boundary is set to $\xi_{in}$ = 0.03 and the outer boundary is set to $\xi_{ex}$ = 0.2. When the mean aggregation degree is $\xi_{m}<0.03$, the encoding (transmission) rate will be improved automatically; when the mean aggregation degree is $\xi_{m}>0.2$, the encoding (transmission) rate will be reduced automatically; when the mean aggregation degree is $0.03\leq \xi_{m}\leq 0.2$, the encoding (transmission) rate will be kept the same.

**Figure 16:**
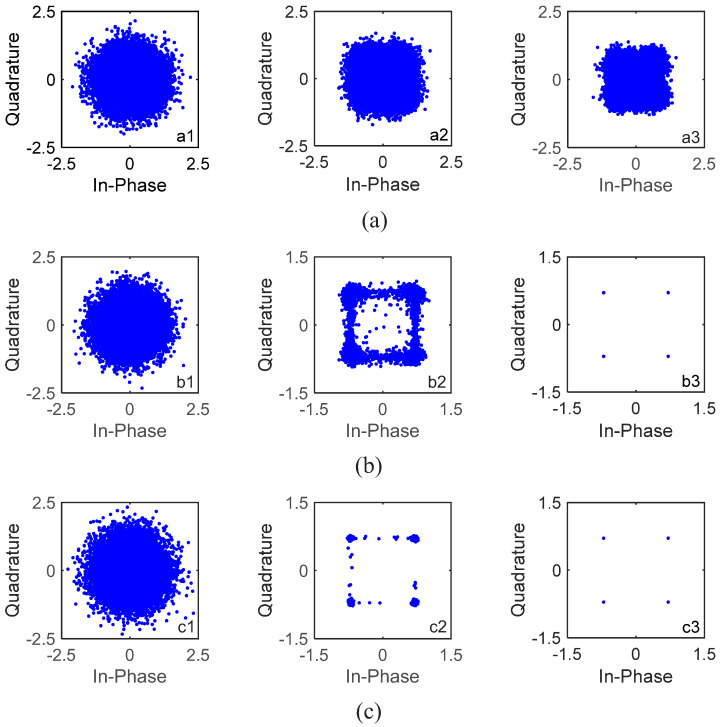
Constellations of the 16 blocks of data with S256 after 0, 1 and 2 iterations for Yellow Sea 1 in Figure 17. (**a1**) Rate-1/4, 0 iteration; (**a2**) rate-1/4, 1 iteration; (**a3**) rate-1/4, 2 iterations; (**b1**) rate-1/8, 0 iteration; (**b2**) rate-1/8, 1 iteration; (**b3**) rate-1/8, 2 iterations; (**c1**) rate-1/16, 0 iteration; (**c2**) rate-1/16, 1 iteration; (**c3**) rate-1/16, 2 iterations.

The calculation of the mean aggregation degree $\xi_{m}$ is shown in Table 3. For Yellow Sea 1, assuming that rate-1/16 convolutional code was used first, after one iteration, the mean aggregation degree was $\xi_{m}$ = 0.002, which was less than 0.03. Therefore, the encoding rate was improved automatically, i.e., the encoding rate was adjusted from rate-1/16 convolutional code to rate-1/8 convolutional code automatically. We can see that, after one iteration, with rate-1/8 convolutional code, the mean aggregation degree was $\xi_{m}$ = 0.0591, which belonged to $\left[0.03,0.2\right]$. Therefore, the encoding rate was kept the same. Assuming that rate-1/4 convolutional code was used first, after one iteration, the mean aggregation degree was $\xi_{m}$ = 0.4442, which was more than 0.2. Therefore, the encoding rate was reduced automatically, i.e., the encoding rate was adjusted from rate-1/4 convolutional code to rate-1/8 convolutional code automatically. As after one iteration with rate-1/8 convolutional code, the mean aggregation degree was $\xi_{m}$ = 0.0591, which belonged to $\left[0.03,0.2\right]$, the encoding rate was kept. The aggregation performance of the 16 blocks of information bits with S256 is shown in Figure 16. From Figure 16b, after one iteration with rate-1/8 convolutional code, the constellation points of the 16 blocks of information bits were obviously clustered.

**Figure 17:**
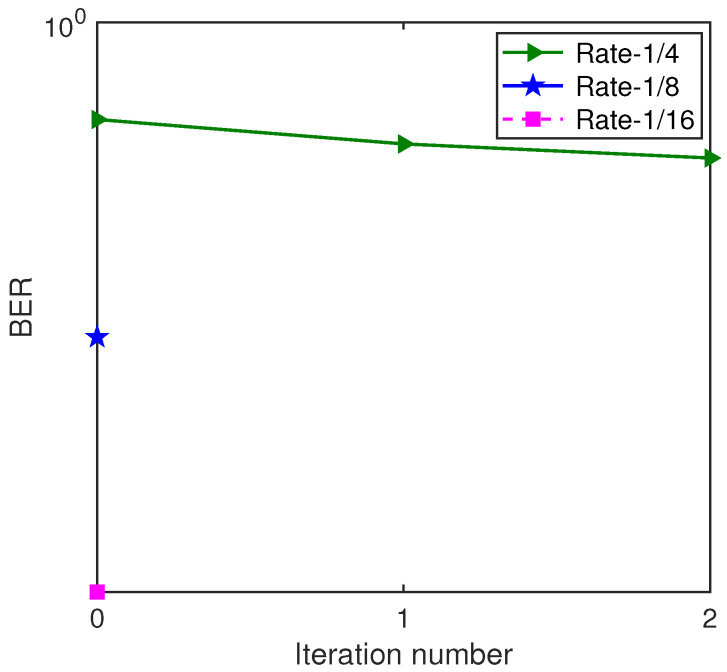
BER performance of the GL turbo system with S256 for Yellow Sea 1.

The BER performance based on the ST scheme and the GL algorithm with S256 for Yellow Sea 1 is shown in Figure 17. We can see that, after one iteration, the decoding with rate-1/4 convolutional code was invalid, and the decodings with rate-1/8 convolutional code and rate-1/16 convolutional code were valid. With rate-1/8 convolutional code, all information bits were correctly decoded after one iteration, and the BER performance was sufficient in meeting the needs for underwater acoustic communications. Therefore, the rate-1/8 convolutional code was kept, which was in keeping with the result from the mean aggregation degree, demonstrating the effectiveness of the GL algorithm and the CA algorithm.

### 5.2. Adaptive Underwater Acoustic Communications with SNR = 13 dB

**Figure 18:**
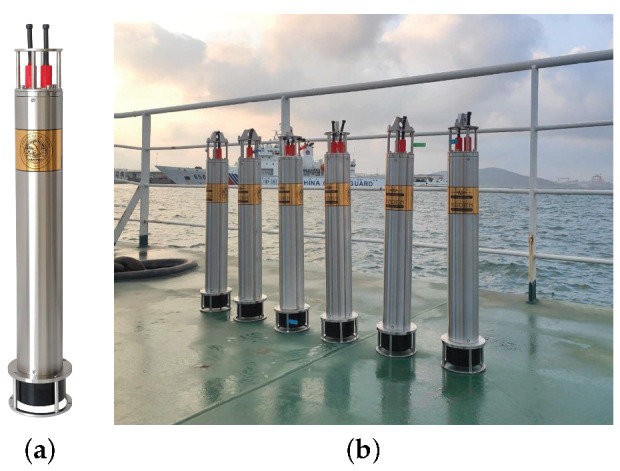
Illustration of underwater acoustic communication machine (Seatrix Modem). (**a**) Single machine; (**b**) multiple machines.

**Figure 19:**
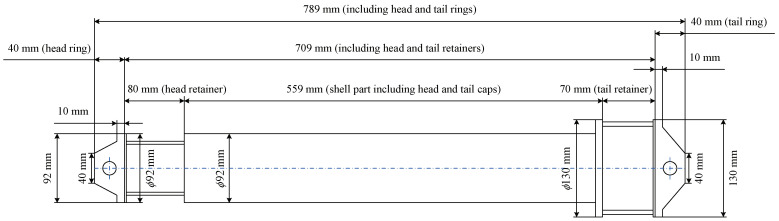
Dimension of underwater acoustic communication machine (Seatrix Modem).

The experimental deployment in the Yellow Sea is shown in Figure 11b. An underwater acoustic communication machine (Seatrix Modem) was used, whose illustration and dimensions are shown in Figure 18 and Figure 19, respectively. The introduction of the machine is listed in Table 4. An SD card was plugged in Seatrix Modem to collect data at the receiver, and the collected data were analyzed by using a computer. The transmitting ship floated away from the receiving ship at a speed of approximately 0.5 m/s. For Yellow Sea 2, rate-1/2 and rate-1/4 convolutional codes were used. S256 was used, and the CP was set to 16 symbols. The transmission rates were 3765 bits/s and 1882 bits/s, respectively, and the corresponding bandwidth efficiencies were 0.94 bps/Hz (rate-1/2) and 0.47 bps/Hz (rate-1/4), respectively. The deployment depths of the transducer and the hydrophone were 4 m and 5 m, respectively. The main goal of the experiment is to demonstrate a successful implementation on modem hardware for the proposed algorithms. As the receiver in Section 5.2 has a higher SNR than the receiver in Section 5.1, higher code rates can be used in Section 5.2. As the communication environments in Section 5.1 and Section 5.2 are similar, their channels are comparable.

**Figure 20:**
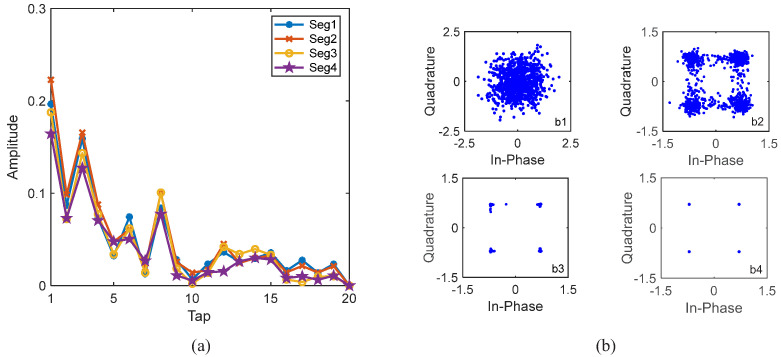
Estimated channels and equalization results of the fourth block with S256 and 1/2 code rate for Yellow Sea 2 in Figure 14. (**a**) The estimated channels of the consecutive four segments after 3 iterations; (**b1**) constellations after 0 iteration; (**b2**) constellations after 1 iteration; (**b3**) constellations after 2 iterations; (**b4**) constellations after 3 iterations.

We used rate-1/2 and rate-1/4 convolutional codes and S256. The BER performance based on the GL algorithm is shown in Table 5. It can be seen that S256 was very effective for underwater acoustic communications with moving transceivers. After only one iteration, all information bits were correctly decoded. Taking the fourth block with rate-1/2 convolutional code in Table 5 as an example, there were four consecutive segments for the fourth data block, and their channels were different due to floating transceivers, i.e., $\alpha_{p}\neq 1$. When turbo equalization was not used, i.e., with 0 iterations, the corresponding channel equalization results are shown in Figure 20(b1), and the constellation points after LMMSE equalization were very scattered. Then, the automatically determined $\alpha_{p}$ was updated. When turbo equalization was performed once, i.e., after one iteration, as shown in Figure 20(b2), the constellation points after LMMSE equalization were still scattered. After three iterations, as shown in Figure 20(b4), the constellation points after LMMSE equalization were ideally condensed together. The corresponding estimated channels of the four segments in Figure 20a were significantly different, where $\alpha_{p}$ = 0.09 after three iterations, demonstrating the time-variation of the channel and the effectiveness of the ST scheme and the GL algorithm. Comparing Figure 15a in Yellow Sea 1 and Figure 20a in Yellow Sea 2, it can be seen that their channel lengths are almost the same. This is because the communication environments are basically the same. We did not show BERs for S512 and W1024, as they basically do not work.

Then, we tested the effectiveness of direct adaptive communications with real underwater acoustic communication machines. The adaptive threshold setting is shown in Table 2. We still used the mean aggregation degree after one iteration to compare the threshold, where the inner boundary is set to $\xi_{in}$ = 0.03 and the outer boundary is set to $\xi_{ex}$ = 0.2. When the mean aggregation degree is $\xi_{m}<0.03$, the encoding (transmission) rate will be improved automatically; when the mean aggregation degree is $\xi_{m}>0.2$, the encoding (transmission) rate will be reduced automatically; when the mean aggregation degree is $0.03\leq \xi_{m}\leq 0.2$, the encoding (transmission) rate will be kept the same.

**Figure 21:**
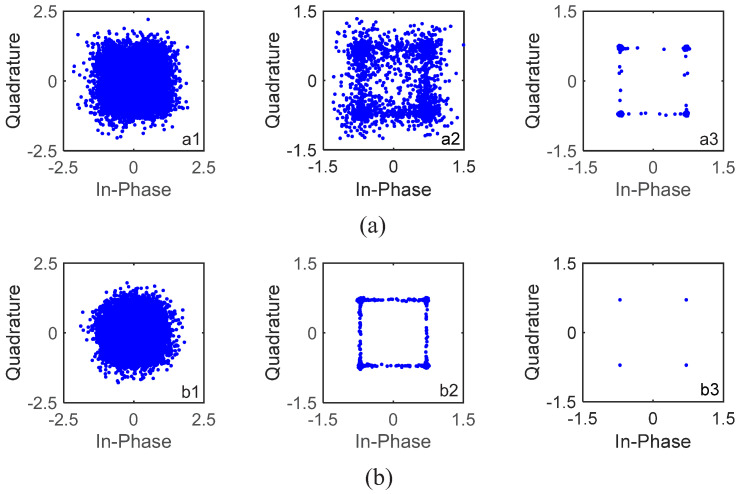
Constellations of the 16 blocks of data with S256 after 0, 1 and 2 iterations for Yellow Sea 2 in Table 5. (**a1**) Rate-1/2, 0 iteration; (**a2**) rate-1/2, 1 iteration; (**a3**) rate-1/2, 2 iterations; (**b1**) rate-1/4, 0 iteration; (**b2**) rate-1/4, 1 iteration; (**b3**) rate-1/4, 2 iterations.

The calculation of the mean aggregation degree $\xi_{m}$ is shown in Table 3. For Yellow Sea 2, assuming that rate-1/4 convolutional code was used first, after one iteration, the mean aggregation degree was $\xi_{m}$ = 0.007, which was less than 0.03. Therefore, the encoding rate was improved automatically, i.e., the channel code was adjusted from rate-1/4 convolutional code to rate-1/2 convolutional code automatically. After one iteration with rate-1/2 convolutional code, the mean aggregation degree was $\xi_{m}$ = 0.041, which belonged to $\left[0.03,0.2\right]$. Therefore, the encoding rate was kept the same. Assuming that rate-1/2 convolutional code was used firstl, after one iteration, the mean aggregation degree was $\xi_{m}$ = 0.041, which belonged to $\left[0.03,0.2\right]$; therefore, the encoding rate was kept. The aggregation performance of the 16 blocks of information bits with S256 for Yellow Sea 2 is shown in Figure 21. From Figure 21a, after one iteration with rate-1/2 convolutional code, the constellation points of the 16 blocks of information bits became obviously clustered.

The BER performance based on the GL algorithm with S256 is shown in Table 5. After one iteration, the decoding performance with rate-1/2 convolutional code was sufficient in meeting the needs for underwater acoustic communications. Therefore, the rate-1/2 convolutional code was kept, which was in keeping with the result from the mean aggregation degree. The experiment demonstrated the effectiveness and practicability of the proposed algorithms in real underwater acoustic communication machines.

From the above simulations and experimental results, we can conclude that the best segment length is $2^{n}$, and should be close to and longer than the channel length, and shorter than a period of the training sequence, where *n* is an integer. Considering the transmission rate and time variation of the channel, S256 is better than S128, S512 and W1024. The two separate experimental results show that, with SNR = 9 dB, the 1/8 code rate is effective; with SNR = 13 dB, the 1/2 code rate is effective. Even if $\alpha_{p}$ = 0.07 and $\alpha_{p}$ = 0.09 ($\alpha_{p}$ can be obtained by calculating the correlation coefficient of the consecutive segments), i.e., the channels of the four segments are weakly correlated, the proposed system is still effective.

## 6. Conclusions

The GL algorithm and the CA algorithm have been proposed to achieve a global accurate channel estimation of each segment and automatic encoding rate adjustment. To improve the estimation and tracking capability of time-varying channels, the ST scheme has been used. For channel estimate fusion of the segments, S256 is the best for practical moving underwater acoustic communications. Even if the channel correlation coefficients of the segments are as low as 0.7 and 0.9, the proposed GL turbo system is still effective. The experimental results demonstrates that a 1/8 code rate is effective at SNR = 9 dB, and a 1/2 code rate is effective at SNR = 13 dB. In the process of iteration, direct adaptive communications based on constellation aggregation have been realized. The experimental results illustrated that the encoding rate can be adjusted automatically among the 1/2 code rate, 1/4 code rate, 1/8 code rate and 1/16 code rate by using the mean aggregation degree decision. Simulations and experimental results have verified the effectiveness of the proposed system.

## Figures and Tables

**Table 1:** Parameters of simulations and experiments.

	Simulation	Yellow Sea 1	Yellow Sea 2
Encoding rate	1/2, 1/4, 1/8, 1/16	1/4, 1/8, 1/16	1/2, 1/4
Power ratio r	0.15:1 to 0.3:1	0.25:1	0.25:1
Segment length	128, 256, 512, 1024 sym	256, 512, 1024 sym	256 sym
CP	128 sym	128 sym	16 sym
1 frame, 1 block	100 blocks, 1024 bits	16 blocks, 1024 bits	16 blocks, 1024 bits
Mapping, system	QPSK, Baseband	QPSK, Single carrier	QPSK, Single carrier
Time dura. of one bit	—	$5\times 10^{-4}$, $10\times 10^{-4}$, $20\times 10^{-4}$ s	$2.5\times 10^{-4}$, $5\times 10^{-4}$ s,
Time dura. of one symbol	—	$2.5\times 10^{-4}$ s	$2.5\times 10^{-4}$ s
Center frequency, Filter	—	12 kHz, Band pass	12 kHz, Band pass
Bandwidth	—	4 kHz	4 kHz
Sampling frequency	—	96 kHz	96 kHz
S256, Transmission rate	—	1333, 667, 333 bits/s	3765, 1882 bits/s
S256, Band. effi. (bps/Hz)	—	0.33 (1/4), 0.17, 0.08	0.94 (1/2), 0.47
Communication distance	—	5.5 km	5.5 km
Transducer depth	—	4 m	4 m
Hydrophone depth	—	4 m	5 m
Relative speed	—	0.5 m/s	0.5 m/s
SNR	−4 dB to 13 dB	approximately 9 dB	approximately 13 dB

**Table 2:** Threshold setting of mean aggregation degree $\xi_{m}$ after one iteration for Yellow Sea 1 and Yellow Sea 2.

$\xi_{m}$< 0.03	Improve the encoding rate
$\xi_{m}$> 0.2	Reduce the encoding rate
0.03 ≤ $\xi_{m}$ ≤ 0.2	Keep the encoding rate

**Table 3:** Calculation of the mean aggregation degree $\xi_{m}$ for Yellow Sea 1 and Yellow Sea 2.

Iteration Number	Yellow Sea 1			Yellow Sea 2	
	Rate-1/4	Rate-1/8	Rate-1/16	Rate-1/2	Rate-1/4
0	0.5546	0.5505	0.5613	0.4719	0.5396
1	0.4442	0.0591	0.002	0.041	0.007
2	0.2953	3.44 × 10${}^{-6}$	3.09 × 10${}^{-6}$	0.0018	1.51 × 10${}^{-6}$

**Table 4:** Introduction of underwater acoustic communication machine (Seatrix Modem).

Communication frequency	9 kHz to 14 kHz (10 kHz to 14 kHz was used)
Communication distance	6000 m with high SNR
Work depth	2000 m
Electrical parameters	Received power consumption 1 w;
	Transmission power consumption 10 w to 60 w;
	Built-in 400 wh rechargeable battery;
	RS-232 interface.
Mechanical parameters	Length 790 mm × Width (Diameter) 130 mm;
	Weight 15 kg (including the battery)

**Table 5:** BER performance of the GL turbo system with S256 at SNR = 13 dB for Yellow Sea 2.

Block Number	Rate-1/2			Rate-1/4		
	Iteration Number			Iteration Number		
	0	1	2	0	1	2
1	8.8%	0	0	0	0	0
2	2.3%	0	0	0	0	0
3	0.7%	0	0	0	0	0
4	3.1%	0	0	0	0	0
5	0	0	0	0	0	0
6	0	0	0	0	0	0
7	0	0	0	0	0	0
8	0	0	0	0	0	0
9	0	0	0	2.0%	0	0
10	0	0	0	0	0	0
11	0	0	0	0	0	0
12	0	0	0	0	0	0
13	0	0	0	0	0	0
14	0	0	0	0	0	0
15	0	0	0	0	0	0
16	0	0	0	0	0	0
Mean	0.9%	0	0	0.1%	0	0

## Data Availability

Not applicable.

## Appendix A

Assuming that the circulant matrix of the impulse response for the channel is $H_{n}$, the transmitted signals are $s_{n}$ and the white Gaussian noise is w. Then, the received signal can be represented as (A1).

(A1) $$\begin{array}{cc} y_{n}\; & \;=\;H_{n}s_{n}+w\;=\;H_{n}\left(rt_{L_{s}}+f_{n}\right)+w\\ \; & \;=\;{\left(\begin{array}{cccccccc} h_{1} & 0 & 0 & \cdots & \cdots & \cdots & \cdots & h_{2}\\ h_{2} & h_{1} & 0 & \ddots & & \ddots & & \vdots \\ \vdots & h_{2} & h_{1} & & \ddots & & \ddots & h_{L_{c}}\\ h_{L_{c}} & \vdots & h_{2} & \ddots & & \ddots & & 0\\ 0 & h_{L_{c}} & \vdots & & \ddots & & \ddots & 0\\ 0 & 0 & h_{L_{c}} & \ddots & & \ddots & & \vdots \\ \vdots & \vdots & 0 & & \ddots & & \ddots & 0\\ 0 & 0 & \vdots & \cdots & \cdots & \cdots & \cdots & h_{1} \end{array}\right)}_{\left(L_{s}+L_{c}-1\right)\times \left(L_{s}+L_{c}-1\right)}{\left(\begin{array}{c} s_{1}\\ s_{2}\\ \vdots \\ s_{L_{s}}\\ 0\\ 0\\ \vdots \\ 0 \end{array}\right)}_{\left(L_{s}+L_{c}-1\right)\times 1}+w\\ \; & \;=\;{\left(\begin{array}{c} h_{1}\\ h_{2}\\ \vdots \\ h_{L_{c}} \end{array}\right)}_{L_{c}\times 1}*{\left(\begin{array}{c} s_{1}\\ s_{2}\\ \vdots \\ s_{L_{s}} \end{array}\right)}_{L_{s}\times 1}+w\\ \; & =h_{n}*s_{n}+w \end{array}.$$

Take elements of $h_{n}$, $s_{n}$, $t_{L_{s}}$,$f_{n}$ and w to build the channel estimator based on the LS algorithm. An element of the impulse response of the channel is denoted by $h_{n}$; an element of the symbol sequence is denoted by $f_{n}$; an element of the periodic training sequence is denoted by $t_{n}$ with a period of *T*; and the superimposed symbol sequence is denoted by $s_{n}\;=\;f_{n}+r\times t_{n}$. As *r* is a constant number, the superimposed symbol sequence can be simplified as $s_{n}\;=\;f_{n}+t_{n}$. Then, an element of the received signal sequence can be expressed as

(A2) $$\begin{array}{c} \begin{array}{cc} y_{n} & \;=\;h_{n}*s_{n}+w_{n}\\ \; & \;=\;\sum_{i\;=\;1}^{L_{c}}f_{\left(n+1-i\right)}h_{i}+\sum_{i\;=\;1}^{L_{c}}t_{\left(n+1-i\right)}h_{i}+w_{n}, \end{array} \end{array}$$

where $w_{n}$ and $L_{c}$ is the white Gaussian noise and the channel length, respectively. Assuming that the mean value of the symbol sequence is 0, and $T\geq L_{c}$, then we can obtain

(A3) $$E\left[y_{n}\right]\;=\;\sum_{i\;=\;1}^{L_{c}}t_{\left(n+1-i\right)}h_{i}.$$

Let $L_{s}\;=\;pT$. We divide $E\left[y_{n}\right]$ into *p* subsegments with a length of *T*, i.e.,

(A4) $$E\left[y_{n}^{}\right]\;=\;E{\left(\begin{array}{c} y_{1}\\ y_{2}\\ \vdots \\ y_{L_{s}} \end{array}\right)}_{L_{s}\times 1}\;=\;E\left(\begin{array}{c} y_{1T}^{}\\ y_{2T}^{}\\ \vdots \\ y_{pT}^{} \end{array}\right),$$

where

(A5) $$E\left[y_{iT}\right]\;=\;{\left[\begin{array}{cccc} t_{iT+1} & t_{iT} & \cdots & t_{iT-L_{c}+2}\\ t_{iT+2} & t_{iT+1} & \cdots & t_{iT-L_{c}+3}\\ \vdots & \vdots & \ddots & \vdots \\ t_{iT+T} & t_{iT+T-1} & \cdots & t_{iT+T-L_{c}+1} \end{array}\right]}_{T\times L_{c}}{\left(\begin{array}{c} h_{1}\\ h_{2}\\ \vdots \\ h_{L_{c}} \end{array}\right)}_{L_{c}\times 1}\overset{\Delta }{\;\;}=\;Ah_{n}.$$

As we use a periodic training sequence with a period of *T*, we can acquire

(A6) $$A\;=\;\left(\begin{array}{cccc} t_{1} & t_{T} & \cdots & t_{T-L_{c}+2}\\ t_{2} & t_{1} & \cdots & t_{T-L_{c}+3}\\ \vdots & \vdots & \ddots & \vdots \\ t_{T} & t_{T-1} & \cdots & t_{T-L_{c}+1} \end{array}\right).$$

It can be seen from (A5) that A is a Toeplitz matrix, and its mean is not related to *i*. A cumulative sum calculation is performed for $E\left[y_{n}\right]$. When *p* is a large value, we can obtain

(A7) $$\frac{1}{p}\sum_{i\;=\;1}^{p}E\left[y_{iT}\right]\;=\;\frac{1}{p}\sum_{i\;=\;1}^{p}\left[y_{iT}\right]\;=\;Ah_{n}.\;$$

When $T\geq L_{c}$, based on the LS algorithm, we can obtain (3).
